# Transanal full-thickness excision for rectal neoplasm: is it advisable to leave the defect open?

**DOI:** 10.1007/s00423-022-02745-9

**Published:** 2023-01-06

**Authors:** J. A. Gracia, M. Elia, E. Cordoba, A. Gonzalo, J. M. Ramirez

**Affiliations:** 1 Department of Surgery, University Hospital of Zaragoza, San Juan Bosco 15, 50009 Saragossa, Spain; 2 Aragon Health Research Institute, San Juan Bosco 13, 50009 Saragossa, Spain

**Keywords:** Rectal tumor, Transanal surgery, Local surgery, Rectal cancer

## Abstract

**Purpose:**

After a full-thickness total wall excision of a rectal tumor, suturing the defect is generally recommended. Recently, due to various contradictory studies, there is a trend to leave the defects open. Therefore, this study aimed to determine whether leaving the defect open is an adequate management strategy compared with suturing it closed based on postoperative outcomes and recurrences.

**Methods:**

A retrospective review of our prospectively maintained database was conducted. Adult patients who underwent transanal surgery for rectal neoplasm in our institution from 1997 to 2019 were analyzed. Patients were divided into two groups: sutured (group A) or unsutured (group B) rectal defect. The primary outcomes were morbidity (early and late) and recurrence.

**Results:**

In total, 404 (239 men) patients were analyzed, 143 (35.4%) from group A and 261 (64.6%) from group B. No differences were observed in tumor size, distance from the anal verge or operation time. The overall incidence of complications was significantly higher in patients from group B, which nearly double the rate of group A. With a mean follow-up of 58 (range, 12–96) months, seven patients presented with a rectal stricture, all of them from group B.

**Conclusions:**

We acknowledge the occasional impossibility of closing the defect in patients who undergo local excision; however, when it is possible, the present data suggest that there may be advantages to suturing the defect closed.

## Introduction

The optimal management for benign rectal neoplasm is endoscopic snare polypectomy; however, for large sessile villous polyps, surgical excision is indicated due to the high risk of malignancy [1]. Additionally, local excision is also the procedure of choice for carefully selected rectal carcinomas. For these lesions, local removal is an adequate treatment only if the tumor has been completely dissected, including the underlying muscularis propria, along with the normal mucosal margin [2, 3].

Transanal endoscopic microsurgery (TEM), introduced by Buess in 1985, was conceived to facilitate the removal of tumors located in the mid and upper rectum under direct vision [4]. With technology and minimal invasive instrument advancements, some other approaches are gaining acceptance as alternatives to TEM for local transanal rectal surgery [5, 6].

Regardless of the selected platform, the generally accepted last step of the technique is to suture the defect based on the traditional rectal surgical criteria. However, rectal wall defect closure is one of the most time-consuming parts of the procedure, representing a real challenge even for skilled surgeons, and is not feasible in some cases [7]. These facts raised doubts about the real benefits of this last step, with surgeons questioning whether leaving the defect open truly results in poor outcomes. In 2002, we conducted the only published randomized study on this topic. No differences in early or late complications were observed whether the defect was closed or left open [8].

Since then, some other observational studies have emerged, but with contradictory results [9]. To date, the debate is more alive due to the widespread implementation of transanal rectal surgery owing to improvements in surgical laparoscopic techniques.

Therefore, this study primarily aimed to analyze our series of transanal local excision of rectal tumors and to compare postoperative complications of patients with sutured with those with defects left open, trying to identify risk factors that could help surgeons make this decision.

## Materials and methods

### Study design

All demographic, preoperative and operative, pathological, and follow-up data of patients with rectal lesions eligible for local surgery from our department since 1997 are prospectively collected.

We performed a retrospective review of our institutional prospective database regarding all patients aged > 18 years treated with transanal full-thickness excision for benign sessile adenomas or early rectal carcinomas. Patients who underwent immediate radical surgery were excluded from this study.

Patients were divided into two groups based on whether the rectal defect was sutured (group A) or left unsutured (group B). The main outcome measures were the incidence of postoperative complications and recurrences. Severity was graded using the Clavien–Dindo classification. For the purpose of the study, only patients with a minimum follow-up of 12 months were analyzed.

### Preoperative evaluation

Preoperative evaluation included a careful history taking, digital rectal examination, and full colonoscopy with biopsy. Rigid proctosigmoidoscopy was performed in all patients to determine the lesion level in the rectum. In this sense, our protocol was established according to Buess et al. [10], who defined the length of peritoneal reflection up to 12 cm anteriorly, 15 cm laterally, and 20 cm posteriorly. According to Najarian et al.’s study in 2005 [11], we avoided including any patient with an upper tumor margin further than 15 cm from the anal verge, regardless of the spatial location in the rectum.

The evaluation also included an endorectal ultrasound and pelvic MRI for patients with biopsy-proven adenocarcinoma since 2003.

All patients were fully informed about the procedure and were included after signing an informed consent.

### Procedure

TEM equipment was indicated for most patients; however, the traditional transanal approach was used for the lowest lesions. Full-thickness excision was performed for all rectal tumors. In 2005, as an improvement of the existing technique [1, 3], an ultrasonic scalpel device from Ethicon (New Jersey, USA) was used [12]. During the early years of TEM at our institution, suturing the defect was the treatment of choice; however, using the results of our randomized study [8], this step was no longer routinely performed, and the decision was based on the surgeon’s preference.

Patients started a normal diet on the first postoperative day and were usually discharged home after tolerating the diet, achieving adequate pain control on oral analgesia, and normal observations of vital signs.

### Follow-up

Patients were followed up at the outpatient clinic underwent anamnesis including the Cleveland clinic fecal incontinence severity score, digital rectal examination, and rigid proctosigmoidoscopy for the first time in 4 weeks, then every 3 months up to the second postoperative year, and at 6- to 12-month intervals thereafter.

### Statistical analysis

Summary data are presented as mean and standard deviation for continuous variables and percentages for discrete variables. To evaluate an increased probability of having an open defect depending on demographic and preoperative characteristics, the odds ratio associated with logistic regression was calculated. To analyze the risk of showing different outcomes depending on open or closed defects, the odds ratio associated with logistic regression was calculated. All statistical tests were two-sided, and a *p*-value of < 0.05 was considered significant. Analysis was performed using R version 4.0.5 (2021–03-31).

## Results

During the study period between January 1997 and December 2019, 404 (239 male) patients were eligible: 143 (35.4%) with defect closed and 261 (64.6%) with defect left open (Table 1). The mean age and size of the resected specimen were 68 (range, 20–92) years and 3.7 (range, 1–11) cm, respectively. Regarding the distance from the anal verge to the upper tumor margin, the overall mean was 9.6 (range, 2–20) cm. The mean distance was significantly higher during the first years, before we began to follow the recommendations of Niajara et al. in 2005 (11 vs. 8.4 cm).

**Table 1:** Patient, tumor, and operative characteristics of open vs. closed defects

	`	CLOSED DEFECT Group A	OPEN DEFECT Group B	OR	*p-*value
	*N* = *404*	*N* = *143*	*N* = *261*		
Mean age, year (SD)	68.0 (11.9)	68.7 (11.9)	67.6 (11.9)	0.99 [0.97;1.01]	0.357
Male	239 (59.2%)	72 (30.1%)	167 (69.9%)	1.75 [1.16;2.65]	0.008
ASA, *n* (%)					
I	71 (17.6%)	27 (38.0%)	44 (62.0%)	Ref	Ref
II	225 (55.7%)	75 (33.3%)	150 (66.7%)	1.23 [0.70;2.13]	0.470
III–IV	108 (26.7%)	41 (38.0%)	67 (62.0%)	1.00 [0.54;1.86]	0.991
Mean height from anal verge, cm (SD)	9.56 (3.86)	9.65 (3.44)	9.51 (4.08)	0.99 [0.94;1.04]	0.733
Anterior location, *n* (%)	103 (25.5%)	34 (33.0%)	69 (67.0%)	1.15 [0.72;1.86]	0.563
Circumferential, *n* (%)	17 (4.21%)	4 (23.5%)	13 (76.5%)	1.77 [0.61;6.58]	0.311
Lateral location, *n* (%)	89 (22.0%)	42 (47.2%)	47 (52.8%)	0.53 [0.33;0.86]	0.010
Posterior location, *n* (%)	195 (48.3%)	63 (32.3%)	132 (67.7%)	1.30 [0.86;1.96]	0.212
Ultrasonic scalpel, *n* (%)	214 (53.0%)	74 (34.6%)	140 (65.4%)	1.08 [0.72;1.62]	0.717
Margin involvement, *n* (%)	20 (4.99%)	5 (25.0%)	15 (75.0%)	1.65 [0.62;5.27]	0.332
Tumor mean size, cm (SD)	3.66 (1.56)	3.61 (1.34)	3.68 (1.67)	1.03 [0.90;1.18]	0.642
Mean operation time, min (SD)	77.7 (44.9)	80.8 (47.6)	76.0 (43.4)	1.00 [0.99;1.00]	0.308
Operation technique:					
TEM	364 (90.1%)	126 (34.6%)	238 (65.4%)	Ref	Ref
Transanal	40 (9.90%)	17 (42.5%)	23 (57.5%)	0.72 [0.37;1.41]	0.329
Preoperative biopsy, *n* (%)					
adenocarcinoma	71 (17.6%)	32 (45.1%)	39 (54.9%)	Ref	Ref
adenoma	333 (82.4%)	111 (33.3%)	222 (66.7%)	1.64 [0.97;2.76]	0.065
Definitive pathology, *n* (%)					
pT1	79 (79.0%)	36 (45.6%)	43 (54.4%)	Ref	Ref
pT2	21 (21.0%)	10 (47.6%)	11 (52.4%)	0.92 [0.35;2.48]	0.868

*ASA* American Society Anesthesiology, *SD* standard deviation

Of the 404 included patients, the tumor was located anteriorly in 103 (25.5%) and posteriorly in 195 (48%) patients. Full-thickness excision was performed in all cases. Regarding dissection, the conventional monopolar scalpel was used in 190 patients (47%), and 214 were operated using the new ultrasonic device. The average operation time was 78 (range, 15–270) min. During the procedure, no unexpected complications occurred; however, the peritoneal cavity was entered in six (1.4%) patients; we could suture the opening in all except one patient, who needed assistance of laparoscopic sutures to close the opening.

When comparing the two groups (Table 1), patients who underwent defect closure (group A) had a higher incidence of female sex and laterally located lesions. No differences were observed in tumor size, mean distance from the anal verge, type of scalpel used, or mean operation time. A non-significant trend was observed to suture the defects when the patient underwent a biopsy of cancer (*p* = 0.06).

Regarding the primary outcome, the overall incidence of postoperative complications was of 27.7% (Table 2), the rate significantly different between the two groups: group B showed nearly double the complication rate of group A. Early postoperative morbidity was recorded in 51 (12.4%) patients. Bleeding was the most common, occurring in 30 (7.4%) patients, 23 of whom were from group B (*p* > 0.05). In seven patients with bleeding, a repeated TEM was required for hemostasis (Clavien–Dindo IIIb). Local sepsis was observed in four (0.9%) patients, one of whom also needed reoperation.

**Table 2:** Postoperative outcomes of open vs. closed defects

	ALL	CLOSED DEFECT Group A	OPEN DEFECT Group B	OR	*p-value*
	*N* = *404*	*N* = *143*	*N* = *261*		
Readmissions, *n* (%)	7 (1.73%)	0 (0.00%)	7 (2.68%)	–	–
Incidence of postoperative complications, *n* (%)	112 (27.7%)	28 (19.6%)	84 (32.2%)	1.94 [1.20;3.21]	0.006
Incidence of early complications (< 30 days), *n* (%)	51 (12.6%)	9 (6.29%)	42 (16.1%)	2.81 [1.38;6.38]	0.004
Postoperative death	1 (0.25%)	0 (0.00%)	1 (0.38%)		
Bleeding	30 (7.44%)	7 (4.90%)	23 (8.85%)	1.85 [0.81;4.84]	0.150
Local infection					
Urinary retention	5 (1.24%)	0 (0.00%)	5 (1.92%)		
Urinary infection	4 (0.99%)	0 (0.00%)	4 (1.53%)		
Unexplained fever	7 (1.73%)	0 (0.00%)	7 (2.68%)		
Postoperative ileus	1 (0.25%)	1 (0.70%)	0 (0.00%)		
Clavien–Dindo:					
I	24 (50.0%)	7 (77.8%)	17 (43.6%)		
II	16 (33.3%)	0 (0.00%)	16 (41.0%)		
IIIb	8 (16.7%)	2 (22.2%)	6 (15.4%)		
Late postoperative complications, *n* (%)	9 (2.23%)	1 (0.70%)	8 (3.07%)	3.99 [0.71;101]	0.132
Fistula	2 (0.50%)	1 (0.70%)	1 (0.38%)	0.55 [0.01;21.4]	0.708
Stricture	7 (1.73%)	0 (0.00%)	7 (2.68%)		
Fecal incontinence	34 (8.42%)	11 (7.69%)	23 (8.81%)	1.15 [0.55;2.54]	0.713
Recurrence, *n* (%)	44 (12.5%)	11 (8.46%)	33 (14.8%)	1.86 [0.93;4.01]	0.081
Adenoma	37 (10.5%)	9 (6.92%)	28 (12.6%)	1.92 [0.90;4.49]	0.091
Carcinoma	7 (1.98%)	2 (1.54%)	5 (2.24%)	1.50 [0.30;11.8]	0.636

^*^Early postoperative morbidity: any complications before 30 days after operation

With a mean follow-up of 58 (range, 12–96) months, seven patients presented with a rectal stricture, all of them from group B (*p* < 0.05) and two ano-vaginal fistulae. Minor fecal incontinence was also recorded in 34 (8.4%) patients. All these complications were detected during the first postoperative year.

During the follow-up, local recurrence occurred in 44 (12.5%) patients (33 from group B and 11 from group A), and the mean duration for the onset of recurrence was 18 months. Recurrence occurred more frequently in group B, but this difference was not significant (*p* = 0.08).

## Discussion

Endoscopic resection is the preferred management strategy for benign colorectal polyps, although removing superficial submucosal invasive cancers is recently accepted [13]. Local rectal surgery remains the standard management of broad-based polyps and selected low-risk rectal or small cancers in patients unfit for major surgery [14, 15]. Local excision can potentially balance acceptable oncological results and very good functional results by avoiding the morbidity of a radical resection [16].

Local excision of rectal tumors significantly improved the resection quality in the mid-1980s, when TEM emerged as a minimally invasive technique [17]. TEM allows full-thickness rectal resection and suture closure of the resultant defects under an excellent view of the entire rectal cavity. However, the considerable upfront cost of TEM instrumentation and difficulties of the technique in the pre-laparoscopic era limited its widespread use, and TEM remained a technique performed at a few reference centers [18].

Recently, the extensive development of laparoscopic and minimally invasive tools is expanding the possibilities of endoluminal surgery [19], and local rectal excision can currently be performed using not only the transanal approach or TEM equipment but also the transanal modified single-port procedure, known as the TAMIS procedure [5], or even robotic surgery [20]. Most postoperative complications associated with local full-thickness rectal excision (with either TEM or TAMIS) can be easily managed and usually occur during the first postoperative month [21–23].

In any case, as these new platforms are more easily accessible and regular laparoscopic instruments can be used, several laparoscopic surgeons have removed rectal tumors trans-anally. They are coping with the technical challenge of closing the surgical defect in the rectal wall after completing the local excision [24].

According to Buess [10], with the TEM equipment, the defect is closed by continuous running suture using a resorbable material. Then, different suturing methods have been described in the literature, such as Endo-GIA staplers [5], intracorporeal running sutures [25], or extracorporeal single suturing with a knot pusher [26].

Obviously, closure of an intraperitoneal perforation is mandatory; however, without peritoneal entry, the benefits of defect closure are still unclear. Thus, understanding the well-vascularized mesorectum provides a good medium for rectal wall regrowth. This concept, combined with the mentioned technical difficulties, are perhaps the reasons why some authors started a scientific debate, maintaining that leaving the defect open has similar outcomes as closing it [27] or even better results [28].

In 2002, we published the only randomized controlled trial on this subject to date. A total of 44 patients were prospectively randomized, 21 sutured and 19 unsutured, and no differences in early or late complications could be demonstrated [8]. However, this study is limited due to the low sample size, and data from the following observational non-randomized studies are contradictory.

On one hand, favorable defect closure was observed, such as that in the Brown et al. [29] single-cohort study that aimed to analyze early complications. With 342 patients included, authors found a significantly lower complication rate in the sutured closed group. They also found that the surgeon’s experience was a factor in suturing the rectal defect. In a multicenter study that also limited to comparing short-term complications, Lee et al. concluded no significant difference between sutured and unsutured defects; however, they recommended a selective approach due to bleeding trend in unsutured defects [30]. A third prospective study, with 53 patients recruited, found that the chance of having grade III postoperative complications was reduced 16-fold in sutured surgical defects [31].

On the other hand, an observational study in 2016 reported that defect closure after a transanal rectal excision was associated with higher morbidity [32]. In a study of 75 patients designed to evaluate peri-operative complications, Hahnloser et al. [9] concluded that the defect could be left unsutured without increasing complications or compromising continence. In this series, the surgeon was unable to suture the defect in approximately 30% of the patients.

In 2017, a meta-analysis that included four studies was published, yielding 489 patients (317 and 182 in the closed and open groups, respectively). This meta-analysis showed no significant difference in the overall morbidity, postoperative local infection, postoperative bleeding, and postoperative reintervention rates between the sutured and unsutured groups [7]. However, the study has several limitations, showing differences in peri-operative management, surgeon’s experience, and operative technique. Moreover, the criteria for defect closure were also different between the included studies.

Nowadays, the mainstream view seems to be that the decision can be safely left to the surgeon’s preference. A recent study [33] described the outcome of 35 patients who underwent TAMIS without closure of the rectal defect over a 6-year period. With an overall morbidity of 14.2%, this study discovered that it is possible to leave the rectal defect open in all transanal excision procedures in the extraperitoneal rectum, regardless of the size and histopathology of the lesion or whether or not neoadjuvant treatment is indicated.

Our study results are, to our knowledge, the largest comparative study of sutured versus unsutured management that include early and late postoperative outcomes, shown an overall rate of complications similar to reported studies [10, 34]; however, the registered incidence of morbidity was significantly higher in the group with defects left open. Most severe complications were from this group, and more patients needed a reintervention. Although our first study [8] found that the unsutured approach is not riskier, supposedly due to the small number of randomized patients, the present data suggest advantages to suturing the defect closed. Our results concur with studies suggesting that leaving the defect open as a routine practice remains controversial, and we attempted to close all defects below the peritoneal reflection [24].

### Limitations

This study is a secondary analysis of our data and has the limitations of being a retrospective study. Results may not be generalizable, because all procedures were performed by highly trained specialist colorectal surgeons at a reference center.

## Conclusions

TEM full-thickness excision provided a low rate of postoperative morbidity and potentially avoided several major abdominal operations. The introduction of the minimally invasive transanal approach has enabled more surgeons to incorporate the technique, contributing to the expansion of surgical local excision worldwide.

We acknowledge the occasional impossibility of closing the defect in patients who undergo a local excision, and the decision of whether to close the defect or not should be made by considering these particular technical issues. If there are no such issues, we highly recommend suturing for defect closure.
